# Faith, Hope, and Therapy: A Qualitative Study to Explore Faith-Based Leaders’ Perceptions of Mental Illness in the Rural South

**DOI:** 10.5888/pcd23.260031

**Published:** 2026-06-04

**Authors:** Shameka L. Cody, Shena Sanchez, Wanda M. Burton, Lilanta J. Bradley, Pamela P. Foster, Sharlene D. Newman

**Affiliations:** 1 Capstone College of Nursing, The University of Alabama, Tuscaloosa, Alabama; 2 Department of Educational Studies, The University of Alabama, Tuscaloosa, Alabama; 3 Department of Community Medicine and Population Health, The University of Alabama, Tuscaloosa, Alabama; 4 University of Alabama Heersink School of Medicine, Bioinformatics Department, Birmingham, Alabama; 5 Alabama Life Research Institute, The University of Alabama, Tuscaloosa, Alabama

## Abstract

**Introduction:**

Faith-based leaders are often the first people contacted by people in rural areas who are experiencing mental health crises, yet some may delay referrals. Public health professionals and mental health providers should therefore understand the perspectives of faith-based leaders on mental health. In this qualitative study, we explored the perceptions of faith-based leaders regarding mental illness and the role of the church in addressing stigma associated with mental illness in the rural South.

**Methods:**

Using purposeful sampling, pastors and ministers (N = 10) were recruited from 3 rural Southern counties. Participants viewed a minidocumentary featuring faith-based leaders discussing their role in addressing mental health issues in the church. After viewing, participants shared their thoughts in focus groups.

**Results:**

Thematic qualitative analysis identified 3 themes: 1) mental health is a broad topic involving various aspects connected to a person’s overall well-being; 2) church and faith-based leaders are unprepared to address mental health stigma among church congregants; and 3) faith-based leaders expressed that they could address mental health needs by building relationships with congregants through listening and having a spirit of discernment.

**Conclusion:**

Future studies with larger samples of faith-based leaders from different religious backgrounds are needed. Integrated spiritual-based and mental health prevention approaches may be beneficial to support faith-based leaders in their role in referring people to treatment.

**Summary**

**What is already known about this topic?**

Faith-based leaders (ie, pastors and ministers) are trusted people in rural communities and play a key role in supporting people experiencing mental health crises. However, assessing how faith-based leaders define and perceive mental illness in the rural South is important.

**What is added by this report?**

Our study provides perspectives of faith-based leaders in the rural South related to their role in addressing the stigma frequently associated with mental illness.

**What are the implications for public health practice?**

Despite the documented scarcity of trained health care providers in rural areas, certain faith-based leaders are uniquely positioned to partner with public and mental health professionals. Such collaborations represent a critical, contemporary approach to improving use of mental health services.

## Introduction

Although mental health disorder rates are similar in urban and rural areas, rural communities face large shortages of trained providers and facilities (1). Some residents rely on primary care for mental health needs, while others travel long distances to the nearest clinic. Access is further limited by high poverty, unemployment, and large numbers of people who are uninsured or underinsured (2). Even when services exist, many rural residents avoid seeking care due to cultural beliefs, stigma, distrust of providers, or fear of disclosure (2). These barriers contribute to untreated or delayed diagnoses, which increase risks for poor physical health, substance use, serious mental illness, and suicide (1,3).

Despite these challenges, many rural residents turn to faith and seek support from pastors and ministers during mental health crises. Religious involvement can foster resilience and positive coping for those experiencing mental illness (4,5). However, some church members interpret mental illness as demonic possession or lack of faith, which reinforces stigma and discourages professional help-seeking. In some communities, discussing mental illness or pursuing treatment is taboo, thus contributing to people with mental illness feeling shame or having a “fix it yourself” mentality (6).

Research is limited on faith-based interventions for rural residents with mental illness (7,8) and on church perceptions of mental illness (6). In a qualitative study of 8 southern California churches, participants described both hospitality toward people with mental illness and barriers such as theological beliefs that may exclude or stigmatize people, including reliance on spiritual solutions over professional care (6). Understanding faith-based leaders’ views on mental illness is important because they often do not refer people to mental health services (9–11), and health professionals rarely refer people with mental health disorders to clergy for support (9). Therefore, we examined faith-based leaders’ views on mental illness in rural Alabama, a state with one of the highest depression rates in the US (12). Focus groups were conducted to address the research questions:

1. How do faith-based leaders perceive mental illness?
2. What are the perceptions of faith-based leaders regarding the role of the church in addressing mental health stigma?

## Methods

In this qualitative study, we used purposeful sampling to recruit faith-based leaders from 3 rural Alabama counties, defined by using US Census Bureau and related rural classification metrics (13,14). Focus group times were scheduled on the basis of participant availability, and recruitment occurred through flyers and word of mouth. Interested leaders contacted a community health worker for a confidential eligibility screening. Focus groups were held at the nearest city hall and facilitated by our research team, which included a qualitative specialist. 

### Study procedures

This study received institutional review board approval. Three focus groups were conducted between October and December 2024. Participants provided written consent and then completed an electronic survey related to mental health stigma and religious support. Afterward, faith-based leaders from 3 rural counties watched a minidocumentary and participated in a 2-hour focus group. For convenience, leaders from 2 counties were combined for the first 2 sessions, and a third session was held with leaders from the remaining county.

On the day of the focus groups, we reviewed the study purpose, addressed questions, and emphasized confidentiality. Participants then watched *Faith, Hope, and Therapy*, a 5-minute university-produced minidocumentary featuring leaders from diverse faiths discussing their roles in mental health. Before viewing the minidocumentary, participants were informed that it was used solely to prompt discussion. After viewing, they were given a few minutes for individual reflection on their own experiences and interpretations before sharing initial thoughts in a group discussion. Three to four academic researchers facilitated each session using a semistructured guide. A follow-up session was held a few weeks later for participants who wished to add or elaborate on ideas. Refreshments and a gift card were provided as incentives.

### Priming

Activating relevant mental concepts before collecting qualitative data helps support better recall and disclosure, which is particularly salient when discussing topics that are associated with stigma (15,16). We used a survey for priming to gather initial insights on mental health and stigma before conducting the focus groups (17). The electronic survey included the modified Mental Health Stigma Survey (18) and the Multidimensional Measurement of Religiousness/Spirituality for Use in Health Research (19). 

The stigma survey included agree or disagree statements such as, “People with mental health disorders have only themselves to blame.” The religious support measure used a 4-point scale asking how much support participants expected from their congregation during difficult situations. Priming helped orient faith-based leaders to the topics and prepared them for discussion.

### Semistructured focus group questions

Focus groups were selected for data collection. Each focus group was led by asking semistructured, open-ended questions. Probes were used to explore spiritual views of mental illness that might help or hinder people seeking help with mental illness. Questions 2 and 3 served as the primary focus for the study.

1. What are your immediate thoughts of the video?
2. What is mental illness?
3. What role does the church play in addressing mental health stigma?

Prompt: What mental health issues do you see?

1. How do you care for your own mental health?
2. Additional prompts included: Is there anything else you feel is important to say on these topics? Do you feel comfortable helping people with their mental health? Did you get any other resources/people involved?

### Qualitative analysis

The data for this study consisted of transcripts from the 3 focus groups, which were analyzed thematically to identify patterns in faith-based leaders’ perspectives. Thematic analysis is a commonly used method in qualitative research that allows researchers to interpret and construct meaning from data based on similarities that participants share (20,21). Guided by an interpretivist paradigm, the second author (S.S.) interpreted the findings based on participants’ descriptions of supporting congregants’ mental well-being.

The first phase of analysis involved identifying and removing inconsistencies in the data by reviewing the transcripts for accuracy and notating initial impressions of the conversations in analytic memos. Following that, the data were uploaded into the ATLAS.ti qualitative software (22), which was used for the entire analysis. The second author (S.S.) developed thematic codes based on the initial reading of the transcripts and applied them during a round of open coding (23), and revised, eliminated, and added codes as appropriate according to patterns identified. A second coding cycle focused on the most salient codes, based on their frequency and depth of discussion. These codes were then grouped into categories, which were constructed into themes supported by relevant literature and survey measures. Trustworthiness was supported through reflexive practice and researcher triangulation (24–26). The second author (S.S.) examined her assumptions when coding and engaged coauthors to avoid isolated analysis. Preliminary findings were reviewed with the team for alignment with theory, literature, and data-collection observations, and no coding disagreements emerged. To protect the identity of participants and their parishioners, all names in this article are pseudonyms and any other identifiers were changed to ensure confidentiality.

## Results

Participants included 10 faith-based leaders (8 pastors and 2 ministers) from Baptist, Methodist, and nondenominational churches across 3 rural Southern counties. All identified as Black or African American; 4 were women and 6 were men. Participants ranged in age from 30 to 79 years. Eight had at least a high school diploma, and 7 were married.

### Perceptions of mental health and the role of the church

The faith-based leaders in this study recognized that mental health is a “really broad” topic, making it difficult to define. One participant described mental health as “the ability to make sound decisions where you can be productive in life.” Despite not having a common definition, the consensus among faith-based leaders in this study was that mental health is tied to a person’s entire well-being, which they believe is made up of their spiritual, physical, social, and financial states.

Additionally, they shared that from their experiences with church members and people they meet, mental health is often indicative of how well or poorly someone is doing in these aspects of their life. That is, they believe that a great deal of people’s mental health struggles often stem from compounding challenges in life (eg, interpersonal, financial, physical), negatively affecting their mental health over time. One faith-based leader elaborated on the ways that unresolved issues can expand and become a larger problem, saying:

Here’s something else that we have to also be reminded of, because all of these things that we kind of overlook leads to some form of mental illness. It starts — nine times out of ten — it normally starts out in a small area, and if we don’t address it, it becomes . . . substance-abuse disorder, substance abuse. All of these things come from an area where we’re not served or being served down here so we can get to where we need to be.

This participant also touched on a shared belief among the others about how the church and faith-based leaders must play a more active role in educating their congregation to diminish the stigma around mental health issues. He goes on to say that the church often does not do enough in this regard, which can also affect the ways that congregants cope with mental health issues, saying the following: “People [are] dealing with mental issues because they’re not educated through the church, and we have to do that from the pulpit, from the teacher’s position.”

In the same vein, another faith-based leader acknowledged that the church can also contribute to people’s mental health challenges with the constant pressure put on them to “live righteously.” Others in the focus group agreed with what she said: “We’re getting into church and we condemn . . . ‘you don’t do this,’ and ‘you don’t do that,’ and ‘the devil going to get you’ . . . you know, this kind of stuff — that’s stressful. . . . We make them sin-conscious by talking about the sin, and that’s a mental thing.” This participant’s awareness of the ways that stress can manifest as a result of religious dogma is notable as it demonstrates an openness to being held accountable for and addressing issues that can come from within the church.

### Desire for better mental health education and training

Despite their desire and intentions to be more involved in supporting the mental well-being of their congregants, faith-based leaders in this study expressed that they often feel unprepared to do so, especially when it comes to more significant issues such as substance abuse, suicide, and other complex comorbidities of mental illness. One participant shared that faith-based leader trainings typically focus more on biblical text and prayer, leaving them insufficiently prepared on how to support people struggling with their mental health, saying, “We’ve been trained a lot to quote scriptures, pray about it, it’ll be okay. But to actually advise, to actually counsel, to actually address a lot of the issues is hard when you’ve not been trained in that capacity.” As a result of challenges they have faced related to not feeling adequately prepared, participants in this study expressed interest in receiving more in-depth training to bolster their ability to recognize the signs of mental health concerns and how to appropriately address them.

Participants also felt that greater resources both for faith-based leaders and church members are needed and that part of their role as ministers involves connecting people to services and resources when the church is not able to provide them. One faith-based leader summed this up by sharing the following anecdote, while others expressed agreement at various points throughout the statement:

The first thing I do is I actually listen intensively to where they are. Share with me how you feel. Let me know what’s going on. You know? Give me — detail me with where you are. And from there, I then begin to think about, okay, can I really address this issue? And there have been several times that I’ve said, ‘Listen, we might need to get you someone that you can actually talk to outside of what I can share from the scripture.’ And I understand that greatly. Because as a pastor, there are some things that I’ve dealt with, and honestly, some things that we deal with a lot now as pastors that you can’t just give me a scripture and tell me you’re going to pray with me about everything. If I know that’s how it is with me, then I must understand that’s how it is with parishioners and the congregation.

In this regard, participants recognized that the church is part of a network of healers in society, alongside doctors, nurses, counselors, and therapists. Thus, they believe that although overall mental well-being can be supported by the church, there are professionals who are appropriately trained and better equipped to treat people who struggle with their mental health. The consensus was that faith-based leaders have a role to play in identifying these issues and helping people get the proper care that they need.

### Importance of a “discerning spirit” and authentic relationships

An area of mental health support where faith-based leaders in this study felt especially confident and capable is in the ways they possess, as one participant puts it, a “discerning spirit,” which is the ability to listen deeply to people’s needs and observe how their actions and demeanor reflect the state of their mental well-being. This finding is related to a previous one about faith-based leaders’ beliefs on the importance of understanding how mental health issues often manifest from acute life challenges that go unaddressed over time. As such, it is important that they are attentive to the unapparent ways that people may be struggling with their mental health. Another participant elaborated on the necessity of having a discerning spirit to be able to get people the support that they need, saying, “We got to pay attention to — like Reverend Janice said — to their actions, to the thing they are not saying. Because a lot of times, we are waiting on them to say ‘I need help.’ But we got to be able to discern that they need help.” Faith-based leaders in this study agreed that deep listening is a key factor in being able to discern that someone is in need of mental health support. As one participant described, deep listening means “you pay attention to body language, you pay attention to words that they use. A lot of people are always *sharing* [emphasis], not necessarily sharing.” The notion of discernment (ie, listening to an inner, God-directed voice) is a common concept among people of faith, which makes it a particularly useful framing for how the participants identify when their members are facing mental health challenges.

Lastly, faith-based leaders in this study expressed that one of the most important ways that the church can meet mental health needs and mitigate stigma is by developing authentic relationships with their congregants and creating an environment where people feel cared for and safe. This begins with meeting people where they are and ensuring that there are other layers of care within the church, as they also agree that all members (not just faith-based leaders) have a role to play in improving mental health outcomes. One participant described the importance of relationships with church members, as others expressed their agreement:

Get a relationship with them and you listen, you know? We need that discerning spirit, but you need an open mind to listen to them and find out what they’re about and talk with it, you know? And I think you have to build there then you go on from there. You know? And it’s not just the pastor’s job. I think we have to build a team. I’ve been working so hard on building teams at church . . . people that can get people and talk and find out what their needs are. . . . You gotta’ start with relationship.

Another faith-based leader, who owned a beauty shop, likened the comfort that her clients felt in her salon to the ways that the church is also a haven for people who are in the midst of mental health struggles. She says that although the primary purpose of churchgoing is to “worship and praise God,” some people may “find more peace just coming in, just sitting in the midst because of the disturbance of what they’re dealing with.” This perspective was shared by other participants, as they believe that the church should be a place where people can feel comforted, validated, and at peace, which can help enhance their mental health.

## Discussion

This study explored rural, Southern, faith-based leaders’ views on mental illness and its associated stigma. A brief documentary prompted discussion, and participants emphasized biologic and stress-related causes, the role of life hardships, and mental health as a reflection of overall coping. Study participants affirmed that mental health is a measure of how well or poorly a person is coping in other areas of their life. Their perspectives are consistent with the concept described by Gautam et al that a person’s entire mental well-being is influenced by numerous variables or determinants (eg, biologic, psychologic, social, and environmental) that interact and influence mental health (27). One participant encouraged greater attention toward determinants that can lead to mental illness, which suggests a holistic approach to promote optimal mental health. These results suggest that faith-based leaders may value a holistic approach to supporting people with mental illness, highlighting the need for deeper partnerships with the health care sector.

Although participants viewed the church as a positive educational resource, they also acknowledged problems with congregants linking mental illness with spiritual sin and causing more stress for people with mental illness. Previous research suggests that faith-based leaders can support mental health well-being but also can reduce well-being through stigma and forced spiritualization (28,29). Our study participants voiced feeling unprepared to address mental health–related crises. Also, participants expressed interest in receiving comprehensive training and resources to help them identify and address signs of mental health challenges and connect church members with mental health services. Similarly, in a study of 109 religious leaders, more than 85% desired training to identify mental health problems, and 80% reported they would refer church members to health care professionals (30). This finding aligns with the literature regarding the knowledge and experiences of faith-based leaders around mental health (4,6) and signifies a need for greater access to more information, training, and education on how to support church members with mental health challenges. Participants voiced that their traditional training was limited to spiritual interventions (eg, scriptures and praying), suggesting the need for training that includes integrated spiritual and scientific mental health interventions. Participants shared a perspective that church should be a place where people feel comfortable, at peace, and able to escape their troubles. Across focus groups of congregants from 8 churches, Lehmann et al found similar findings outlining the role of church members in providing a nonjudgmental stance, providing a sense of belonging, and creating a positive experience for people with mental illness (6). Together, findings suggest that training in basic active listening skills could be useful for empowering faith-based leaders and congregation members to provide this sense of hospitality for people with mental illness.

### Limitations

This study has several limitations. The small sample size restricts the generalizability of findings to broader groups of faith-based leaders. Although purposeful sampling further limits generalizability, it was appropriate for exploring perceptions of mental illness within the population. Because some religious traditions are associated with higher levels of mental health stigma, future research should include larger and more diverse samples of faith leaders across multiple denominations. Randomized purposeful sampling may also yield broader insights into how leaders view mental illness and the church’s role in addressing stigma.

### Implications for research and public health

Faith-based leaders in the rural South viewed mental health as part of overall well-being but felt undertrained to address it. Future work should examine how they conceptualize mental health within holistic care and identify training needs that support crisis recognition, stigma reduction, and referral pathways. Partnerships between faith communities and mental health professionals can strengthen literacy, integrate spiritual practices with evidence-based care, and produce shared toolkits to guide support. Additional research is needed on how leaders manage their own mental health, as this may influence their ability to guide others.

Given that many rural residents confide in trusted faith leaders during crises, these leaders can help parishioners access mental health care without stigma. Collaborations between faith communities and mental health providers can bridge gaps in education and treatment. Engaging leaders across diverse religious backgrounds may extend support to people who do not regularly attend church.

### Conclusion

Although rates of mental health disorders are similar in urban and rural settings, rural residents often face poorer outcomes due to limited provider access and persistent stigma. In many rural communities, people turn to pastors or ministers before seeking professional care. Despite their influence, faith-based leaders in the rural South reported feeling unprepared to address mental health needs because of limited training. Our findings highlight the importance of integrated spiritual and mental health prevention training to help leaders identify signs of mental illness and make appropriate referrals. They also underscore the need for partnerships between mental health providers and faith-based leaders to correct misconceptions, reduce stigma, and create trusted pathways for connecting people with professional mental health services.

Faith-based leaders would benefit from culturally responsive mental health training, and our pilot work shows strong demand. We have begun to pilot this training and have found that faith leaders are craving such training. At the policy level, state and county systems should formally integrate faith-based organizations into rural mental health planning and referral networks. Even modest investments in standardized referral protocols could improve early identification, reduce stigma, and expand access to care through trusted community leaders.
